# Supplementary material for: Non-necroptotic MLKL function damages mitochondria and promotes hematopoietic stem cell aging
Source: Nat Commun. 2026 Apr 6;17:2798. doi: 10.1038/s41467-026-71060-4 (PMC13053712; doi:10.1038/s41467-026-71060-4)
Supplement: Supplementary file 1 — Supplementary Information [file 41467_2026_71060_MOESM1_ESM.pdf]

Supplementary Information

Non-necroptotic MLKL function damages mitochondria and promotes hematopoietic stem cell aging

Yuta Yamada, Jinjing Yang, Akiho Saiki-Tsuchiya, Yuji Watanabe, Shuhei Koide, Shin Murai, Yuriko Sorimachi, Yu Fukuda, Kenta Sumiyama, Hiroshi Sagara, Hiroyasu Nakano, Keiyo Takubo, Atsushi Iwama, and Masayuki Yamashita

Supplementary Fig. 1	Preferential and transient activation of MLKL in HSCs upon inflammation.
Supplementary Fig. 2	Inflammation-induced active MLKL impairs HSC function.
Supplementary Fig. 3	The RIPK3-MLKL axis limits HSC function.
Supplementary Fig. 4	MLKL impairs HSC function after 5-FU-induced replication stress.
Supplementary Fig. 5	MLKL impairs HSC function after serial transplantation and promotes ineffective hematopoiesis.
Supplementary Fig. 6	MLKL impairs HSC function during aging.
Supplementary Fig. 7	Minor impact of MLKL on the HSC transcriptome and chromatin accessibility.
Supplementary Fig. 8	MLKL impairs mitochondrial function without affecting reactive oxygen species and autophagy in HSCs.
Supplementary Table 1	Reagents and materials used in this study.

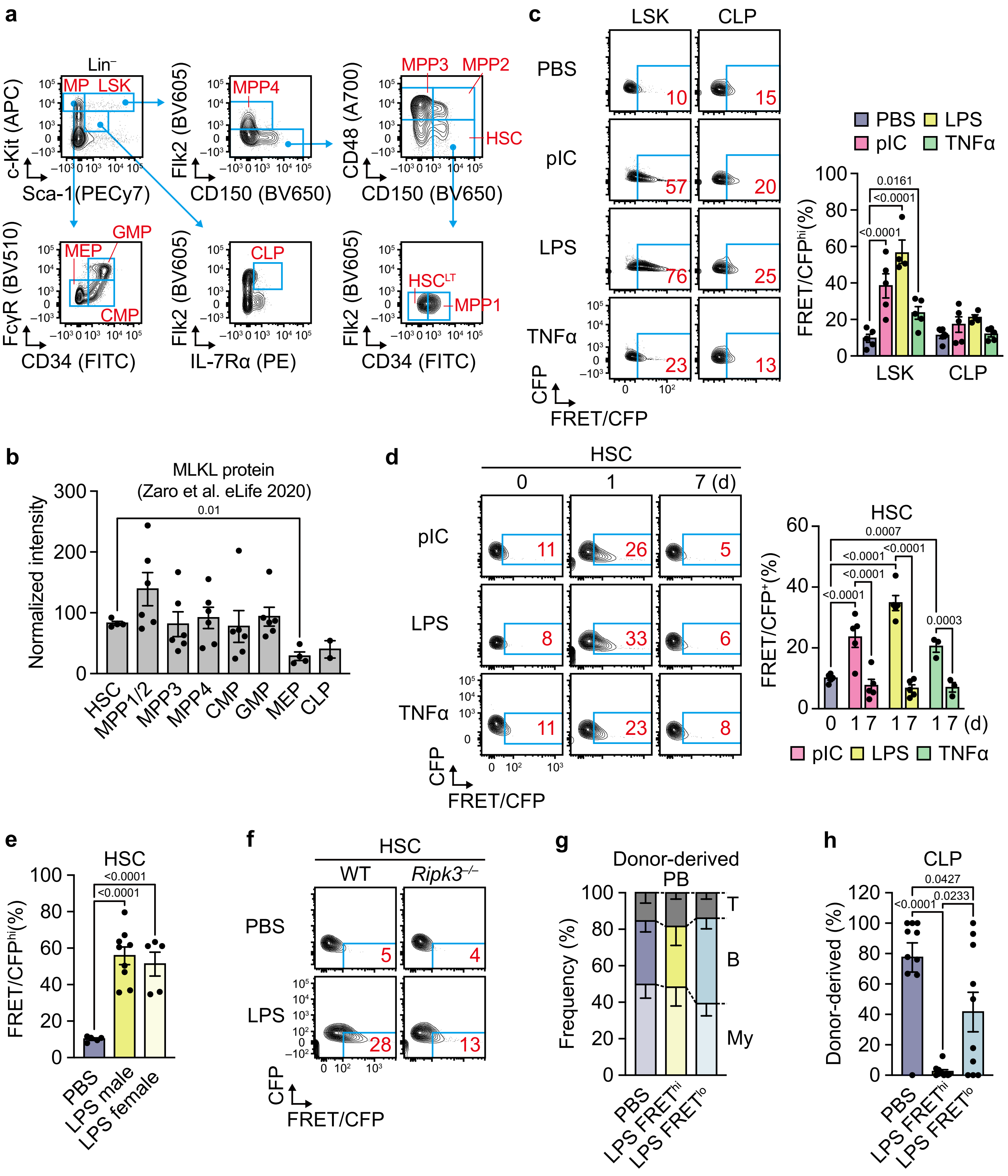
a
Lin⁻
MP
LSK
c-Kit (APC)
Sca-1 (PECy7)
MPP4
Flk2 (BV605)
CD150 (BV650)
MPP3
MPP2
HSC
CD48 (A700)
CD150 (BV650)
MEP
GMP
CMP
FcγR (BV510)
CD34 (FITC)
CLP
Flk2 (BV605)
IL-7Rα (PE)
HSCLT
MPP1
Flk2 (BV605)
CD34 (FITC)
b
MLKL protein
(Zaro et al. eLife 2020)
0.01
Normalized intensity
HSC
MPP1/2
MPP3
MPP4
CMP
GMP
MEP
CLP
c
LSK
CLP
PBS
pIC
LPS
TNFα
10
15
57
20
76
25
23
13
CFP
FRET/CFP
PBS
LPS
pIC
TNFα
0.0161
<0.0001
<0.0001
FRET/CFPhi(%)
LSK
CLP
d
HSC
0
1
7 (d)
pIC
LPS
TNFα
11
26
5
8
33
6
11
23
8
CFP
FRET/CFP
HSC
0.0007
<0.0001
<0.0001
<0.0001
<0.0001
0.0003
FRET/CFP+(%)
0
1 7
1 7
1 7 (d)
pIC
LPS
TNFα
e
HSC
<0.0001
<0.0001
FRET/CFPhi(%)
PBS
LPS male
LPS female
f
HSC
WT
Ripk3−/−
PBS
LPS
5
4
28
13
CFP
FRET/CFP
g
Donor-derived
PB
T
B
My
Frequency (%)
PBS
LPS FREThi
LPS FRETlo
h
CLP
0.0427
<0.0001
0.0233
Donor-derived (%)
PBS
LPS FREThi
LPS FRETlo

Supplementary Fig. 1 | Preferential and transient activation of MLKL in HSCs upon inflammation. **a**, Gating strategies used to identify BM common myeloid progenitors (CMP), granulocyte/monocyte progenitors (GMP), megakaryocyte/erythroid progenitors (MEP), common lymphoid progenitors (CLP), and early stem and progenitor populations (long-term HSC [HSC^{LT}] and multipotent progenitors [MPP1, MPP2, MPP3, and MPP4]) in mice. **b**, MLKL protein expression in BM immature hematopoietic cells obtained via reanalysis of mass spectrometry data shown by Zaro et al[36] (n = 4 HSC, 6 MMP1+2, 6 MPP3, 6 MPP4, 6 CMP, 6 GMP, 4 MEP, and 2 CLP biological replicates). **c,d**, Representative flow cytometry plots and frequencies of FRET/CFP^{hi} LSK cells and CLPs in SMART-Tg mice ± pIC, LPS, and TNF-α at 16 h (**c**) (n = 4 mice in the LPS-treated group and 5 mice/other group; two experiments) and FRET/CFP^{hi} HSCs in SMART-Tg mice ± pIC, LPS, and TNF-α at the indicated time points (**d**) (n = 3 mice in the TNF-α-treated 1 d and 7 d groups and 5 mice/other group; two experiments). **e**, Frequency of FRET/CFP^{hi} HSCs in male and female SMART-Tg mice ± LPS at 16 h. (n = 5 PBS, 9 LPS male, and 5 LPS female mice; two experiments). **f,** Representative flow cytometry plots of FRET/CFP^{hi} HSCs in WT and *Ripk3*$^{-/-}$ SMART-Tg mice ± LPS at 16 h. **g,h,** Donor-derived PB lineage distribution (**g**) (n = 10 PBS-treated, 7 LPS-treated FRET/CFP^{hi}, and 12 LPS-treated FRET/CFP^{lo} HSC recipients; two experiments) and donor chimerism in BM CLPs (**h**) (*n* = 10 recipients/group; two experiments) at 4 months post-transplantation in recipients of BM FRET/CFP^{hi} and FRET/CFP^{lo} HSCs ± LPS. My, myeloid. Data are mean ± s.e.m.; statistical significance was determined using Welch and Brown-Forsythe test (**b**), one-way ANOVA (**e,h**), and two-way ANOVA (**c,d**,**g**) with the two-stage linear step-up procedure of Benjamini, Krieger, and Yekutieli, with exact *P* values shown.

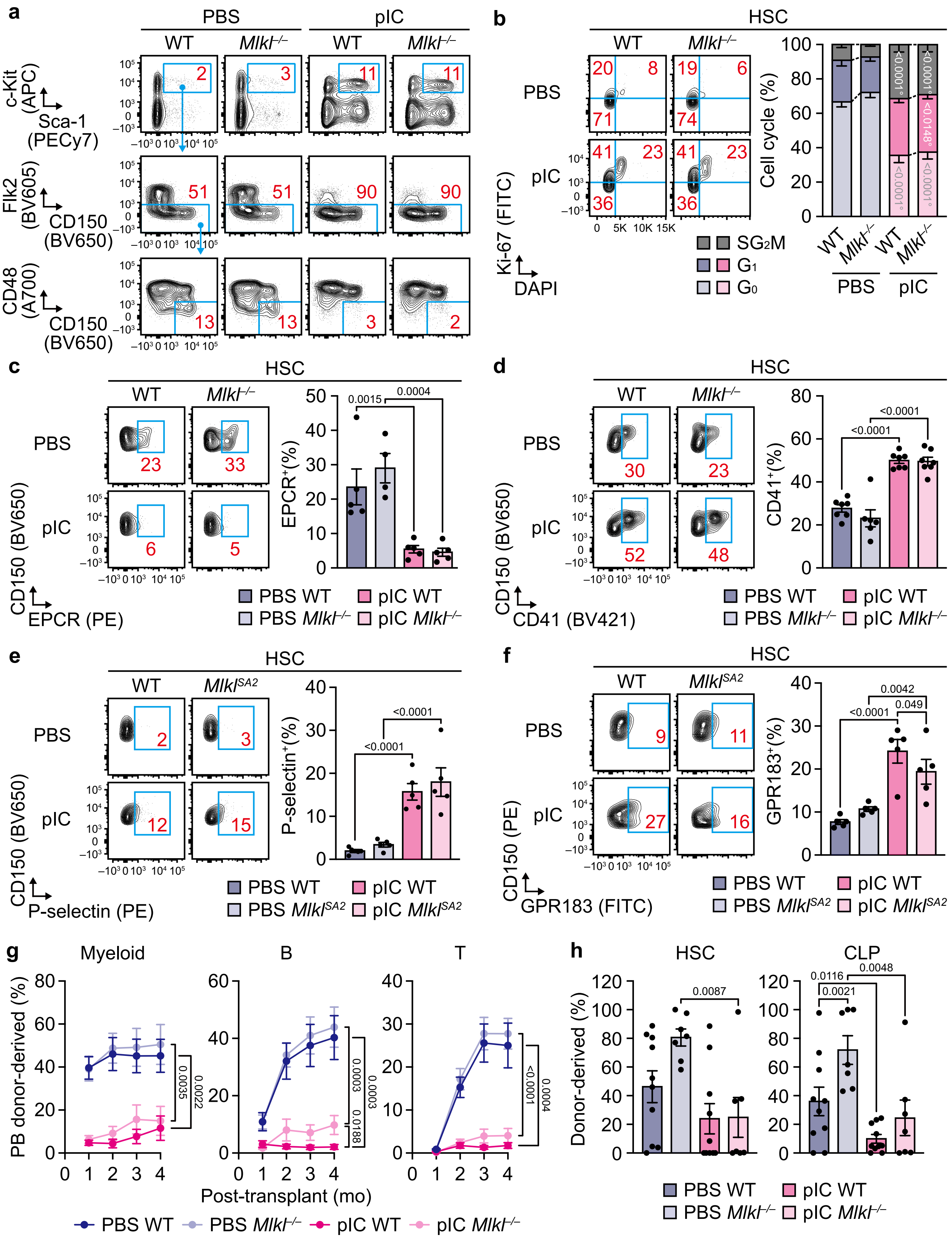
a
PBS
pIC
WT
Mlkl−/−
c-Kit (APC)
Sca-1 (PECy7)
Flk2 (BV605)
CD150 (BV650)
CD48 (A700)
2
3
11
11
51
51
90
90
13
13
3
2
b
HSC
PBS
pIC
Ki-67 (FITC)
DAPI
20
8
71
19
6
74
41
23
36
41
23
36
SG2M
G1
G0
Cell cycle (%)
<0.0001
<0.0148
<0.0001
<0.0001
c
HSC
EPCR (PE)
23
33
6
5
EPCR+(%)
0.0015
0.0004
PBS WT
PBS Mlkl−/−
pIC WT
pIC Mlkl−/−
d
HSC
CD41 (BV421)
30
23
52
48
CD41+(%)
<0.0001
<0.0001
e
HSC
WT
MlklSA2
P-selectin (PE)
2
3
12
15
P-selectin+(%)
<0.0001
<0.0001
PBS MlklSA2
pIC MlklSA2
f
HSC
GPR183 (FITC)
CD150 (PE)
9
11
27
16
GPR183+(%)
0.0042
0.049
<0.0001
g
Myeloid
B
T
PB donor-derived (%)
Post-transplant (mo)
0.0035
0.0022
0.0003
0.0003
0.0188
<0.0001
0.0004
h
HSC
CLP
Donor-derived (%)
0.0087
0.0116
0.0021
0.0048

Supplementary Fig. 2 | Inflammation-induced active MLKL impairs HSC function. a, Representative flow cytometry plots of BM HSCs in WT and *Mlkl*$^{-/-}$ mice ± pIC. **b**, Representative flow cytometry plots and cell cycle distribution of BM HSCs in WT and *Mlkl*$^{-/-}$ mice ± pIC (*n* = 8 mice/group; three experiments). **c**,**d,** Representative flow cytometry plots and frequencies of BM EPCR^{+} HSCs (**c**) (n = 4 mice in the PBS-treated *Mlkl*$^{-/-}$ group and 5 mice/other group; two experiments) and BM CD41^{+} HSCs (**d**) (n = 6 mice in the PBS-treated *Mlkl*$^{-/-}$ group and 7 mice/other group; three experiments) in WT and *Mlkl*$^{-/-}$ mice ± pIC. **e,f,** Representative flow cytometry plots and frequencies of BM P-selectin^{+} HSCs (**e**) (*n* = 5 mice/group; three experiments) and BM GPR183^{+} HSCs (**f**) (*n* = 5 mice/group; three experiments) in WT and *Mlkl*^{SA2}mice ± pIC. **g**, Donor chimerism in PB myeloid, B, and T cells in recipients of WT and *Mlkl*$^{-/-}$ BM HSCs ± pIC (n = 14 PBS-treated WT, 12 PBS-treated *Mlkl*$^{-/-}$, 14 pIC-treated WT, and 13 pIC-treated *Mlkl*$^{-/-}$ HSC recipients; two experiments). **h,** Donor chimerism in BM HSCs and CLPs at 4 months post-transplantation in recipients of BM HSCs from WT and *Mlkl*$^{-/-}$ mice ± pIC (n = 10 PBS-treated WT, 7 PBS-treated *Mlkl*$^{-/-}$, 10 pIC-treated WT, and 7 pIC-treated *Mlkl*$^{-/-}$ HSC recipients; two experiments). Data are mean ± s.e.m.; statistical significance was determined using one-way (**e–f,h**) and two-way ANOVA (**b–d,g**) with the two-stage linear step-up procedure of Benjamini, Krieger, and Yekutieli, with exact *P* values shown; °, versus PBS.

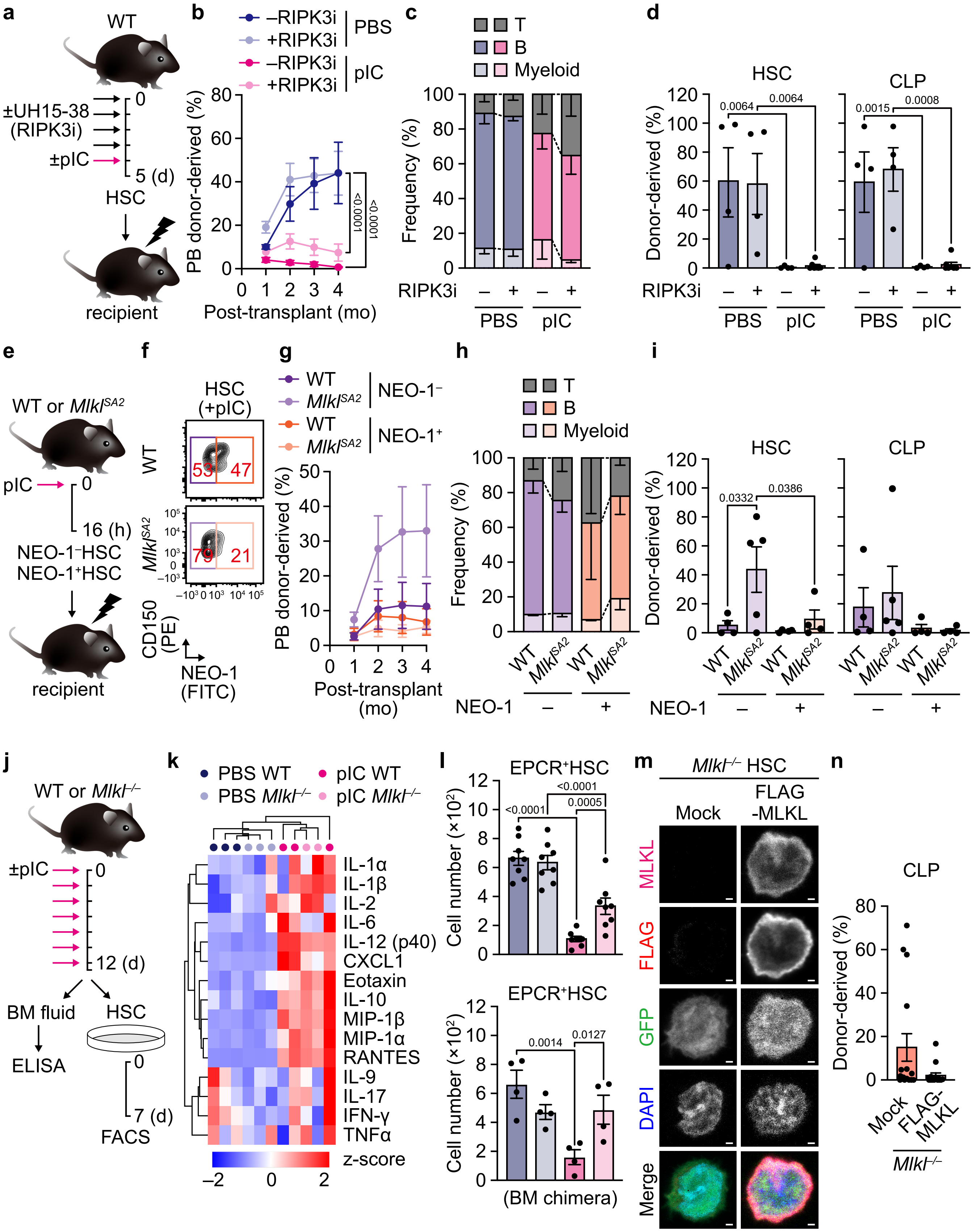
a
WT
±UH15-38
(RIPK3i)
±pIC
0
5 (d)
HSC
recipient
b
–RIPK3i
+RIPK3i
PBS
–RIPK3i
+RIPK3i
pIC
PB donor-derived (%)
Post-transplant (mo)
<0.0001
<0.0001
c
T
B
Myeloid
Frequency (%)
RIPK3i
PBS
pIC
d
HSC
CLP
Donor-derived (%)
0.0064
0.0064
0.0015
0.0008
RIPK3i
PBS
pIC
e
WT or Mlkl^SA2
pIC
0
16 (h)
NEO-1⁻HSC
NEO-1⁺HSC
recipient
f
HSC
(+pIC)
WT
Mlkl^SA2
53
47
79
21
CD150
(PE)
NEO-1
(FITC)
g
WT
Mlkl^SA2
NEO-1⁻
WT
Mlkl^SA2
NEO-1⁺
PB donor-derived (%)
Post-transplant
(mo)
h
T
B
Myeloid
Frequency (%)
WT
Mlkl^SA2
NEO-1
i
HSC
CLP
Donor-derived (%)
0.0332
0.0386
NEO-1
j
WT or Mlkl⁻/⁻
±pIC
0
12 (d)
BM fluid
HSC
ELISA
0
7 (d)
FACS
k
PBS WT
PBS Mlkl⁻/⁻
pIC WT
pIC Mlkl⁻/⁻
IL-1α
IL-1β
IL-2
IL-6
IL-12 (p40)
CXCL1
Eotaxin
IL-10
MIP-1β
MIP-1α
RANTES
IL-9
IL-17
IFN-γ
TNFα
z-score
–2
0
2
l
EPCR⁺HSC
Cell number (×10²)
<0.0001
<0.0001
0.0005
EPCR⁺HSC
0.0014
0.0127
(BM chimera)
m
Mlkl⁻/⁻ HSC
Mock
FLAG
-MLKL
MLKL
FLAG
GFP
DAPI
Merge
n
CLP
Donor-derived (%)
Mock
FLAG-
MLKL
Mlkl⁻/⁻

Supplementary Fig. 3 | The RIPK3-MLKL axis limits HSC function. a–d, Engraftment potential of BM HSCs ± pIC and RIPK3 inhibitor (RIPK3i). Shown are experimental design (**a**), PB donor chimerism (**b**) (n = 5 PBS-treated, 5 PBS/RIPK3i-treated, 8 pIC-treated, and 9 pIC/RIPK3i-treated HSC recipients; two experiments), donor-derived lineage distribution at 4 months (**c**) (n = 4 PBS-treated, 5 PBS/RIPK3i-treated, 4 pIC-treated, and 7 pIC/RIPK3i-treated HSC recipients; two experiments), and donor chimerism in BM HSCs and CLPs at 4 months (**d**) (n = 7 recipients in the pIC/RIPK3i-treated group and 4 recipients/other group; two experiments). **e–i,** Engraftment potential of pIC-treated BM WT and $Mlkl^{-/-}$ NEO-1^{+}/NEO-1^{-} HSCs. Shown are experimental design (**e**), sorting gates (**f**), PB donor chimerism (**g**) (n = 4 WT/NEO-1^{-}, 5 $Mlkl^{SA2}$/NEO-1^{-}, 5 WT/NEO-1^{+}, and 4 $Mlkl^{SA2}$/NEO-1^{+} HSC recipients; one experiment), donor-derived lineage distribution at 4 months (**h**) (n = 2 WT/NEO-1^{-}, 4 $Mlkl^{SA2}$/NEO-1^{-}, 2 WT/NEO-1^{+}, and 3 $Mlkl^{SA2}$/NEO-1^{+} HSC recipients; one experiment), and donor chimerism in BM HSCs and CLPs at 4 months (**i**) (n = 5 recipients in the $Mlkl^{SA2}$/NEO-1^{-} group and 4 recipients/other group; one experiment). **j**, Experimental design for repeated pIC injections (7×pIC). **k**, Normalized inflammatory cytokine levels in WT and $Mlkl^{-/-}$ BM ± 7×pIC (n = 2 mice in the pIC-treated $Mlkl^{-/-}$ group and 3 mice/other group; one experiment). **l**, EPCR^{+} HSC numbers after 7-day culture of HSCs from WT and $Mlkl^{-/-}$ mice (*n* = 8 pools of 500 cells/group; two experiments) and BM chimera (*n* = 4 pools of 500 cells/group; one experiment) ± 7×pIC. **m,** Representative immunofluorescence images of $Mlkl^{-/-}$ HSCs ± FLAG-MLKL (scale bars, 1 µm). **n,** Donor chimerism in BM CLPs at 4 months post-transplantation of $Mlkl^{-/-}$ HSCs ± FLAG-MLKL (n = 16 Mock and 15 FLAG-MLKL HSC recipients; three experiments). Data are mean ± s.e.m.; statistical significance was determined using unpaired two-tailed Student's t-test (**n**) and one-way (**i,l**) and two-way ANOVA (**b–d,g**) with the two-stage linear step-up procedure of Benjamini, Krieger, and Yekutieli, with exact *P* values shown.

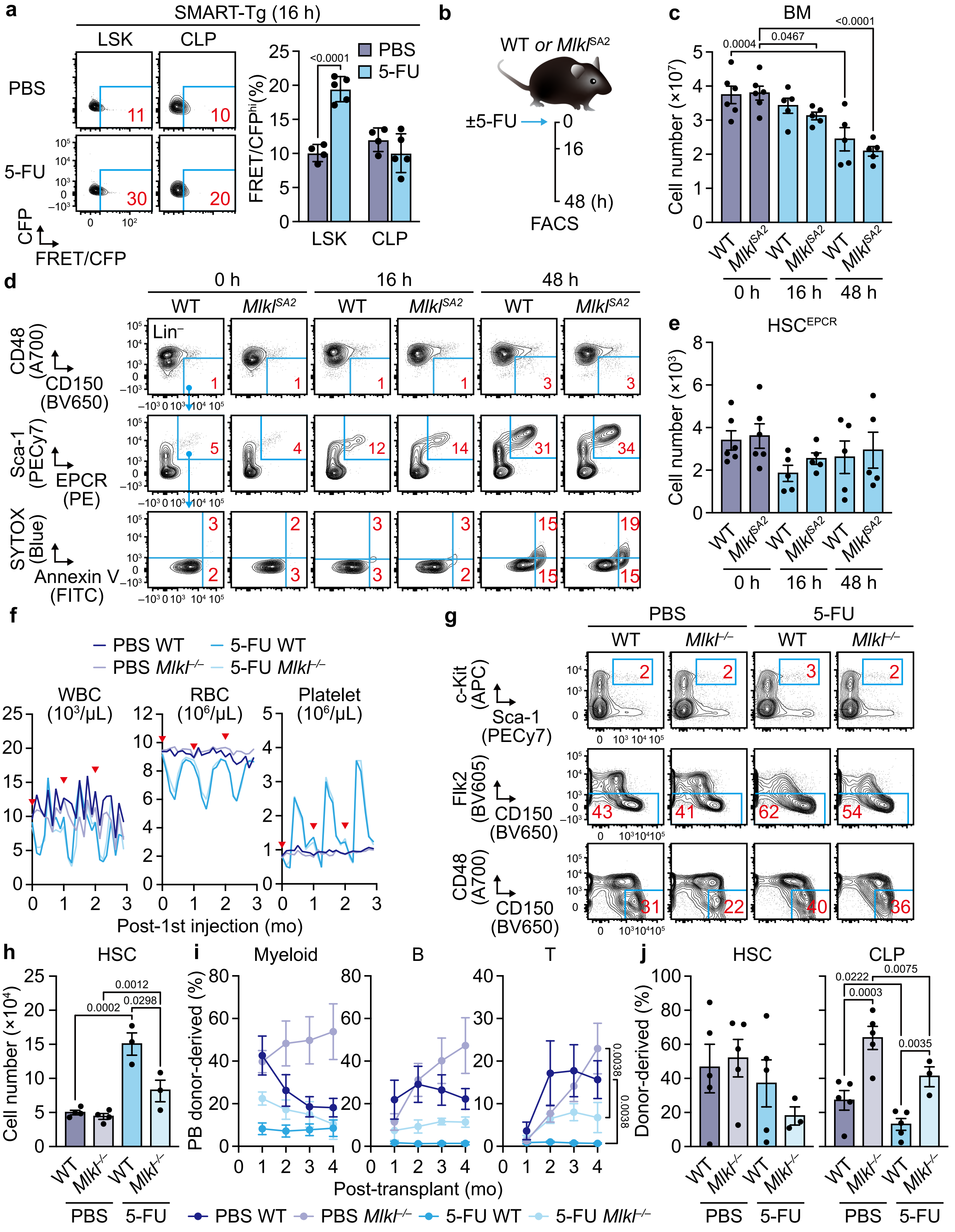

a
SMART-Tg (16 h)
LSK
CLP
PBS
5-FU
11
10
30
20
CFP
FRET/CFP
FRET/CFP^{hi}(%)
<0.0001
PBS
5-FU
LSK
CLP
b
WT or $Mlkl^{SA2}$
±5-FU
0
16
48 (h)
FACS
c
BM
0.0004
0.0467
<0.0001
Cell number (×10^7)
WT
$Mlkl^{SA2}$
0 h
16 h
48 h
d
0 h
16 h
48 h
WT
$Mlkl^{SA2}$
Lin$^-$
CD48 (A700)
CD150 (BV650)
Sca-1 (PECy7)
EPCR (PE)
SYTOX (Blue)
Annexin V (FITC)
1
1
1
1
3
3
5
4
12
14
31
34
3
2
3
3
15
19
2
3
3
2
15
15
e
HSC^{EPCR}
Cell number (×10^3)
WT
$Mlkl^{SA2}$
0 h
16 h
48 h
f
PBS WT
PBS $Mlkl^{-/-}$
5-FU WT
5-FU $Mlkl^{-/-}$
WBC (10^3/μL)
RBC (10^6/μL)
Platelet (10^6/μL)
Post-1st injection (mo)
g
PBS
5-FU
WT
$Mlkl^{-/-}$
c-Kit (APC)
Sca-1 (PECy7)
2
2
3
2
Flk2 (BV605)
CD150 (BV650)
43
41
62
54
CD48 (A700)
CD150 (BV650)
31
22
40
36
h
HSC
Cell number (×10^4)
0.0012
0.0002
0.0298
WT
$Mlkl^{-/-}$
PBS
5-FU
i
Myeloid
B
T
PB donor-derived (%)
0.0038
0.0038
Post-transplant (mo)
PBS WT
PBS $Mlkl^{-/-}$
5-FU WT
5-FU $Mlkl^{-/-}$
j
HSC
CLP
Donor-derived (%)
0.0222
0.0075
0.0003
0.0035
WT
$Mlkl^{-/-}$
PBS
5-FU

Supplementary Fig. 4 | MLKL impairs HSC function after 5-FU-induced replication stress. a, Representative flow cytometry plots and frequencies of FRET/CFP^{hi} LSK cells and CLPs in SMART-Tg mice ± 1×5-FU at 16 h (n = 4 PBS-treated and 5 5-FU-treated mice; two experiments). **b–e,** Acute 5-FU-induced changes in HSC death and absolute cell numbers in WT and *Mlkl*^{SA2} mice (n = 6 mice/genotype in the 0 h groups and n = 5 mice/other group; two experiments). Shown are experimental design (**b**), BM cellularity (**c**), representative flow cytometry plots (**d**), and absolute numbers of BM HSCs in WT and *Mlkl*^{SA2} mice ± 1×5-FU at indicated time points (**e**). Because functional HSCs exhibit markedly reduced c-Kit^{+} expression early after 5-FU exposure, EPCR was used to enrich HSCs as described previously[71]. **f,** PB white blood cell (WBC), red blood cell (RBC), and platelet count changes in WT and *Mlkl*$^{-/-}$ mice ± 3×5-FU (n = 4 mice/group; one experiment). Mean values of each group are depicted. Arrowheads indicate 5-FU injections. **g**, Representative flow cytometry plots of BM HSCs in WT and *Mlkl*$^{-/-}$ mice ± 3×5-FU. **h**, Absolute numbers of BM HSCs in WT and *Mlkl*$^{-/-}$ mice ± 3×5-FU (n = 4 PBS-treated WT, 4 PBS-treated *Mlkl*$^{-/-}$, 3 5-FU-treated WT, and 3 5-FU-treated *Mlkl*$^{-/-}$ mice; one experiment). **i,** Donor chimerism in PB myeloid, B, and T cells in recipients of WT and *Mlkl*$^{-/-}$ BM HSCs ± 3×5-FU (n = 4 mice in the 5-FU-treated *Mlkl*$^{-/-}$ group and 5 mice/ other group; one experiment). **j**, Donor chimerism in BM HSCs and CLPs at 4 months post-transplantation of WT and *Mlkl*$^{-/-}$ BM HSCs ± 3×5-FU (n = 3 mice in the 5-FU-treated *Mlkl*$^{-/-}$ group and 5 mice/other group; one experiment). Data are mean ± s.e.m. except when indicated; Statistical significance was determined using one-way (**h**) and two-way ANOVA (**a,c,e,i,j**) with the two-stage linear step-up procedure of Benjamini, Krieger, and Yekutieli, with exact *P* values shown.

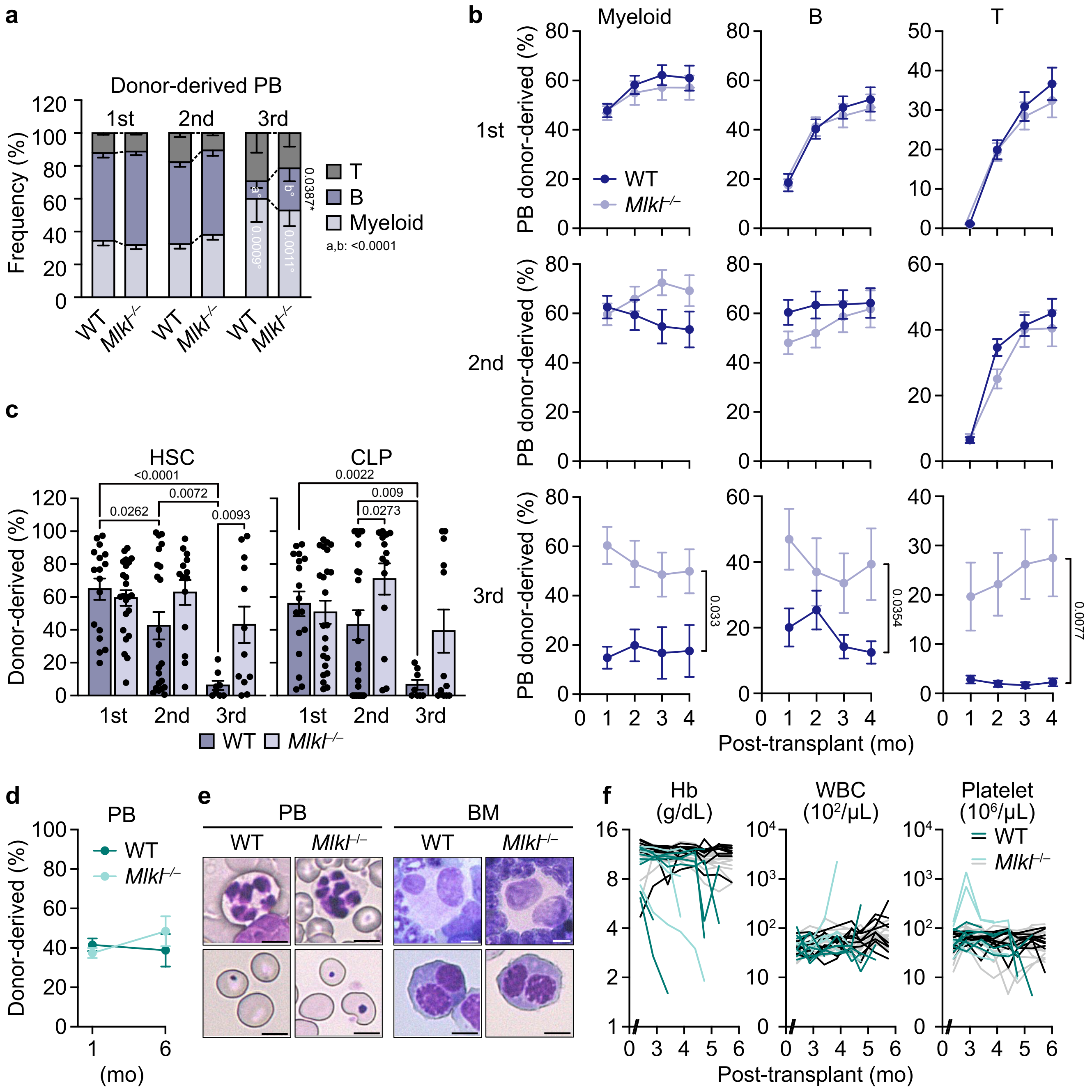

a
Donor-derived PB
1st
2nd
3rd
Frequency (%)
T
B
Myeloid
a,b: <0.0001
0.0387*
0.0009°
0.001°
WT
Mlkl−/−
b
Myeloid
B
T
1st
2nd
3rd
PB donor-derived (%)
WT
Mlkl−/−
0.033
0.0354
0.0077
Post-transplant (mo)
c
HSC
CLP
Donor-derived (%)
<0.0001
0.0262
0.0072
0.0093
0.0022
0.009
0.0273
1st
2nd
3rd
WT
Mlkl−/−
d
PB
Donor-derived (%)
WT
Mlkl−/−
(mo)
e
PB
BM
WT
Mlkl−/−
f
Hb (g/dL)
WBC (10²/μL)
Platelet (10⁶/μL)
WT
Mlkl−/−
Post-transplant (mo)

Supplementary Fig. 5 | MLKL impairs HSC function after serial transplantation and promotes ineffective hematopoiesis. **a**, Donor-derived PB lineage distribution at 4 months post-primary (n = 19 WT and 23 *Mlkl*$^{-/-}$ recipients; five experiments), -secondary (n = 25 WT and 20 *Mlkl*$^{-/-}$ recipients; five experiments), and -tertiary (n = 9 WT and 13 *Mlkl*$^{-/-}$ recipients; five experiments) recipients of BM WT and *Mlkl*$^{-/-}$ HSCs. **b,** Donor chimerism in PB myeloid, B, and T cells in primary (n = 19 WT and 23 *Mlkl*$^{-/-}$ recipients; five experiments), secondary (*n* = 25 mice/group; five experiments), and tertiary (n = 19 WT and 16 *Mlkl*$^{-/-}$ recipients; five experiments) recipients of WT and *Mlkl*$^{-/-}$ BM HSCs. **c**, Donor chimerism in BM HSCs and CLPs at 4 months post-primary (n = 17 WT and 23 *Mlkl*$^{-/-}$ recipients; five experiments), -secondary (n = 22 WT and 14 *Mlkl*$^{-/-}$ recipients; five experiments), and -tertiary (n = 8 WT and 12 *Mlkl*$^{-/-}$ recipients; five experiments) transplantation of BM WT and *Mlkl*$^{-/-}$ HSCs. **d**, PB donor chimerism in recipients of *RUNX1S291fs*-transduced WT and *Mlkl*$^{-/-}$ BM HSCs at 1 month and 6 months post-transplantation (n = 17 WT and 19 *Mlkl*$^{-/-}$ recipients at 1 month; n = 9 WT and 15 *Mlkl*$^{-/-}$ recipients at 6 months; one experiment). **e**, Representative images of May-Grünwald-Giemsa staining showing hypersegmented neutrophils and nucleated RBCs in PB, as well as hypolobulated micromegakaryocytes and multinucleated erythroblasts in BM (scale bars, 10 μm). **f**, PB cell count changes in recipients of *RUNX1S291fs*-transduced WT and *Mlkl*$^{-/-}$ BM HSCs (n = 17 WT and 19 *Mlkl*$^{-/-}$ recipients; one experiment). Values from individual mice are shown and those for moribund mice are highlighted in green. Plt, platelet. Data are mean ± s.e.m. except when indicated; Statistical significance was determined using two-way ANOVA (**a–d**) with the two-stage linear step-up procedure of Benjamini, Krieger, and Yekutieli, with exact *P* values shown; *, versus WT; °, versus 1st.

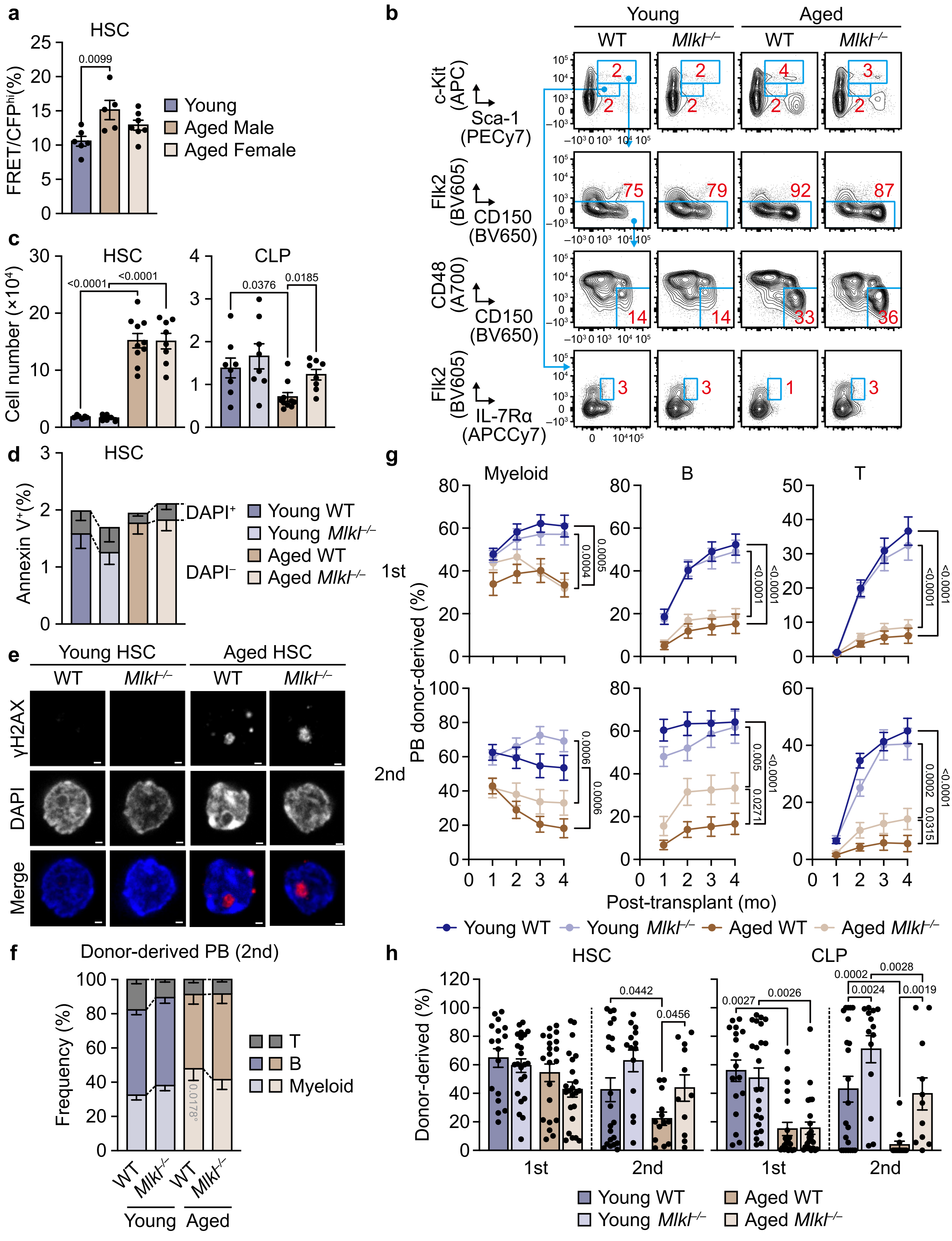

a
HSC
FRET/CFPhi(%)
0.0099
Young
Aged Male
Aged Female
b
Young
Aged
WT
Mlkl−/−
c-Kit (APC)
Sca-1 (PECy7)
Flk2 (BV605)
CD150 (BV650)
CD48 (A700)
IL-7Rα (APCCy7)
c
HSC
CLP
Cell number (×10⁴)
<0.0001
0.0376
0.0185
d
HSC
Annexin V⁺(%)
DAPI⁺
DAPI⁻
Young WT
Young Mlkl−/−
Aged WT
Aged Mlkl−/−
e
Young HSC
Aged HSC
γH2AX
DAPI
Merge
f
Donor-derived PB (2nd)
Frequency (%)
T
B
Myeloid
Young
Aged
g
Myeloid
B
T
1st
2nd
PB donor-derived (%)
Post-transplant (mo)
h
HSC
CLP
Donor-derived (%)
1st
2nd

Supplementary Fig. 6 | MLKL impairs HSC function during aging. a, Frequencies of FRET/CFP^{hi} HSCs in young, aged male, and aged female SMART-Tg mice. (n = 6 young, 5 aged male, and 7 aged female mice; two experiments). **b**, Representative flow cytometry plots of BM HSCs and CLPs in WT and *Mlkl*$^{-/-}$ mice ± aging. **c**, Absolute numbers of BM HSCs and CLPs in WT and *Mlkl*$^{-/-}$ mice ± aging (n = 10 mice in the aged WT group and 8 mice/other group; five experiments). **d**, Frequencies of BM WT and *Mlkl*$^{-/-}$ Annexin V^{+} HSCs ± aging (n = 13 young WT, 14 young *Mlkl*$^{-/-}$, 23 aged WT, and 24 aged *Mlkl*$^{-/-}$ mice; four experiments). **e**, Representative immunofluorescence images for γH2A.X in BM WT and *Mlkl*$^{-/-}$ HSCs ± aging (scale bars, 1 μm). **f–h**, Engraftment potential of BM WT and *Mlkl*$^{-/-}$ HSCs ± aging. Donor-derived PB lineage distribution at 4 months post-secondary transplantation (**f**) (n = 25 young WT, 20 young *Mlkl*$^{-/-}$, 17 aged WT, and 17 aged *Mlkl*$^{-/-}$ recipients; five experiments), donor chimerism in PB myeloid, B, and T cells in primary (n = 19 young WT, 23 young *Mlkl*$^{-/-}$, 23 aged WT, and 24 aged *Mlkl*$^{-/-}$ recipients; five experiment) and secondary (n = 25 young WT, 25 young *Mlkl*$^{-/-}$, 19 aged WT, and 19 aged *Mlkl*$^{-/-}$ recipients; five experiment) recipients (**g**), and donor chimerism in BM HSCs and CLPs at 4 months post-primary (n = 17 young WT, 23 young *Mlkl*$^{-/-}$, 21 aged WT, and 23 aged *Mlkl*$^{-/-}$ recipients; five experiment) and -secondary (n = 22 young WT, 14 young *Mlkl*$^{-/-}$, 13 aged WT, and 11 aged *Mlkl*$^{-/-}$ recipients; five experiments) transplantation (**h**). Data are mean ± s.e.m.; statistical significance was determined using one-way ANOVA (**a**), Welch and Brown-Forsythe test (**c,d**), and two-way ANOVA (**d,f–h**) with the two-stage linear step-up procedure of Benjamini, Krieger, and Yekutieli, with exact *P* values shown; °, versus young.

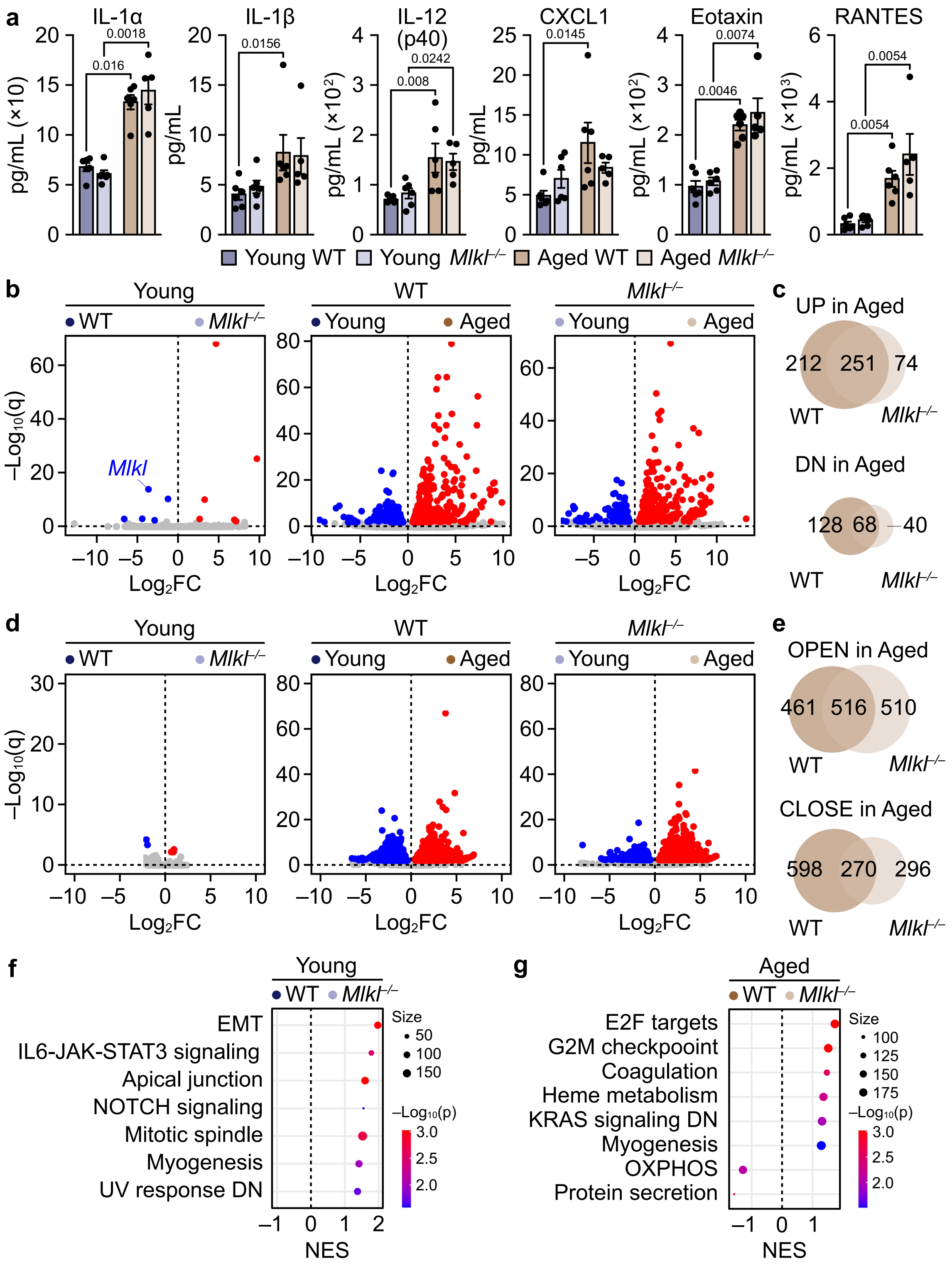
a
IL-1α
IL-1β
IL-12 (p40)
CXCL1
Eotaxin
RANTES
pg/mL (×10)
pg/mL
pg/mL (×10²)
pg/mL (×10³)
0.0018
0.016
0.0156
0.0242
0.008
0.0145
0.0074
0.0046
0.0054
0.0054
Young WT
Young Mlkl–/–
Aged WT
Aged Mlkl–/–
b
Young
WT
Mlkl–/–
Young
Aged
Mlkl
–Log10(q)
Log2FC
c
UP in Aged
212
251
74
WT
Mlkl–/–
DN in Aged
128
68
40
d
e
OPEN in Aged
461
516
510
CLOSE in Aged
598
270
296
f
EMT
IL6-JAK-STAT3 signaling
Apical junction
NOTCH signaling
Mitotic spindle
Myogenesis
UV response DN
Size
50
100
150
–Log10(p)
3.0
2.5
2.0
NES
g
Aged
E2F targets
G2M checkpooint
Coagulation
Heme metabolism
KRAS signaling DN
Myogenesis
OXPHOS
Protein secretion
125
175

Supplementary Fig. 7 | Minor impact of MLKL on the HSC transcriptome and chromatin accessibility. **a**, Absolute concentration of age-related inflammatory cytokines (n = 5 in the aged *Mlkl*$^{-/-}$ group and 6 mice/other group; three experiments). **b**, Volcano plot showing differentially expressed genes (DEGs) in young *Mlkl*$^{-/-}$ versus young WT HSCs, aged WT versus young WT HSCs, and aged *Mlkl*$^{-/-}$ versus young *Mlkl*$^{-/-}$ HSCs. **c**, Venn diagrams showing the number of DEGs in aged WT versus young WT HSCs and aged *Mlkl*$^{-/-}$ versus young *Mlkl*$^{-/-}$ HSCs. **d**, Volcano plot showing DARs in young *Mlkl*$^{-/-}$ versus young WT HSCs, aged WT versus young WT HSCs, and aged *Mlkl*$^{-/-}$ versus young *Mlkl*$^{-/-}$ HSCs. **e**, Venn diagrams showing the number of DARs in aged WT versus young WT HSCs and aged *Mlkl*$^{-/-}$ versus young *Mlkl*$^{-/-}$ HSCs. **f,g,** Gene set enrichment analyses with hallmark gene sets. Shown are significantly enriched gene sets in young *Mlkl*$^{-/-}$ versus young WT HSCs (**f**) and aged *Mlkl*$^{-/-}$ versus aged WT HSCs (**g**). Data are mean ± s.e.m. in (**a**); Statistical significance was determined using Kruskal-Wallis test with the two-stage linear step-up procedure of Benjamini, Krieger, and Yekutieli, with exact *P* values shown.

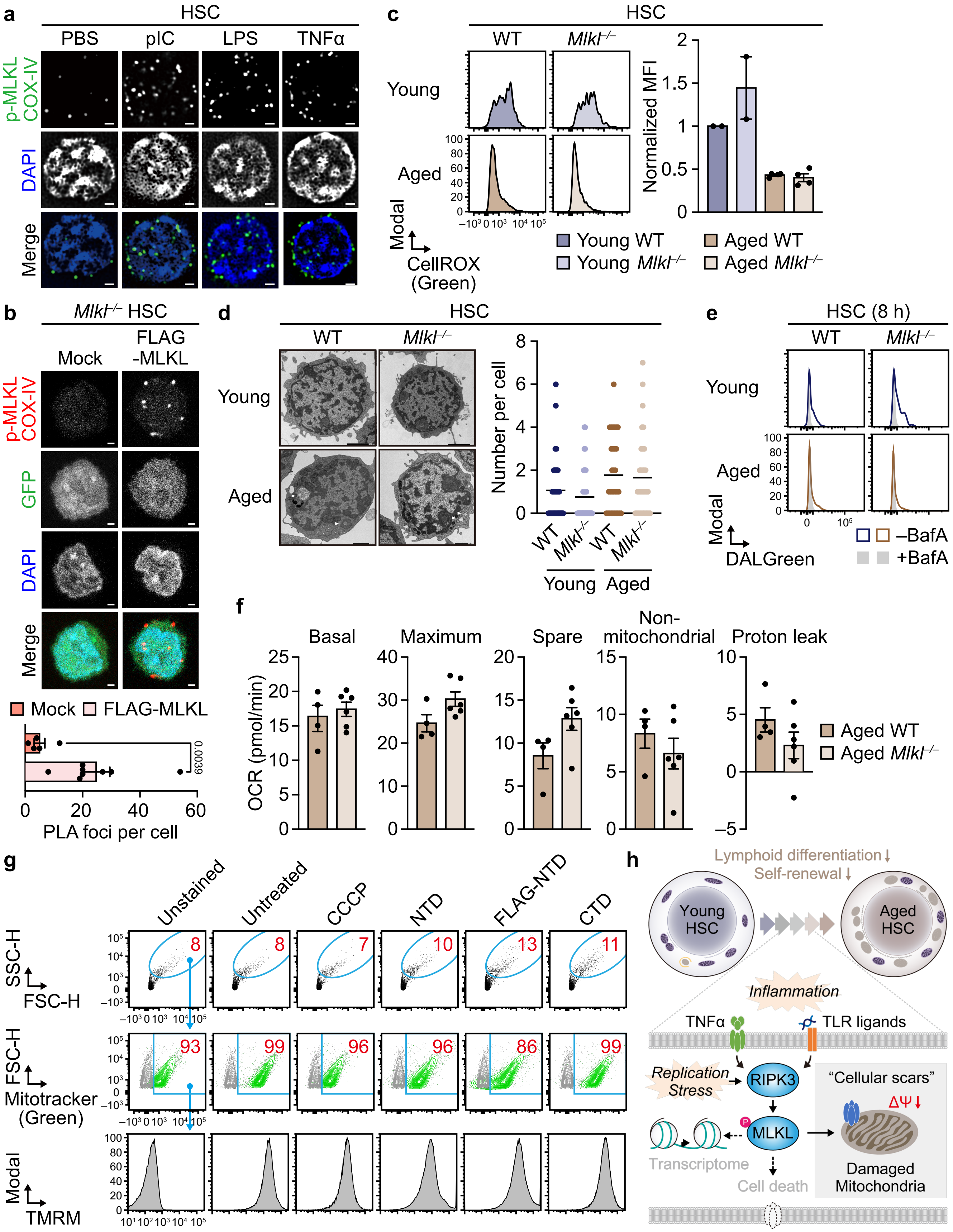

a
HSC
PBS
pIC
LPS
TNFα
p-MLKL COX-IV
DAPI
Merge
b
Mlkl−/− HSC
Mock
FLAG-MLKL
p-MLKL COX-IV
GFP
DAPI
Merge
Mock
FLAG-MLKL
0.0039
PLA foci per cell
c
HSC
WT
Mlkl−/−
Young
Aged
Modal
CellROX (Green)
Normalized MFI
Young WT
Young Mlkl−/−
Aged WT
Aged Mlkl−/−
d
HSC
WT
Mlkl−/−
Young
Aged
Number per cell
WT
Mlkl−/−
WT
Mlkl−/−
Young
Aged
e
HSC (8 h)
WT
Mlkl−/−
Young
Aged
Modal
DALGreen
–BafA
+BafA
f
Basal
Maximum
Spare
Non-mitochondrial
Proton leak
OCR (pmol/min)
Aged WT
Aged Mlkl−/−
g
Unstained
Untreated
CCCP
NTD
FLAG-NTD
CTD
SSC-H
FSC-H
FSC-H
Mitotracker (Green)
Modal
TMRM
h
Lymphoid differentiation↓
Self-renewal↓
Young HSC
Aged HSC
Inflammation
TNFα
TLR ligands
Replication Stress
RIPK3
MLKL
Transcriptome
Cell death
"Cellular scars"
ΔΨ↓
Damaged Mitochondria

Supplementary Fig. 8 | MLKL impairs mitochondrial function without affecting reactive oxygen species and autophagy in HSCs. a, PLA for p-MLKL (S345) and COX-IV in WT and *Mlkl*$^{-/-}$ BM HSCs ± pIC, LPS, and TNF-α (scale bars, 1 μm). **b,** PLA for p-MLKL (S345) and COX-IV in BM *Mlkl*$^{-/-}$ HSCs ± FLAG-MLKL. Shown are representative images (scale bars, 1 μm) and the number of PLA foci (n = 5 Mock and 8 FLAG-MLKL HSCs; two experiments). **c**, Reactive oxygen species in WT and *Mlkl*$^{-/-}$ BM HSCs ± aging. Shown are representative flow cytometry plots and normalized geometric mean fluorescent intensity (MFI) of CellROX (n = 2 young WT, 2 young *Mlkl*$^{-/-}$, 4 aged WT, and 4 aged *Mlkl*$^{-/-}$ mice; two experiments). **d**, Autophagosome in WT and *Mlkl*$^{-/-}$ BM HSCs ± aging. Shown are representative electron microscopy images (scale bars, 2 μm for young and 1 μm for aged HSCs) and the number of autophagosome per HSC (n = 33 young WT, 37 young *Mlkl*$^{-/-}$, 31 aged WT, and 32 aged *Mlkl*$^{-/-}$ HSCs; two experiments). **e**, Autophagy flux in WT and *Mlkl*$^{-/-}$ BM HSCs ± aging after 8-h culture without cytokines. Shown are representative flow cytometry plots of DALGreen staining ± bafilomycin A (BafA). **f**, Mitochondrial respiration measured by Seahorse metabolic flux analyses. Shown are basal respiration, maximum respiration, spare respiration capacity, non-mitochondrial respiration, and proton leaks in aged WT and *Mlkl*$^{-/-}$ BM HSCs (n = 4 WT and 6 *Mlkl*$^{-/-}$ mice; one experiment). **g,** Representative flow cytometry plots of isolated mitochondria ± recombinant MLKL variants (20 μM) or CCCP (50 μM) stained with TMRM and Mitotracker Green. Mitotracker-unstained control (gray) was used to define Mitotracker-positive mitochondrial fractions. **h,** Model for aging of HSCs via non-necroptotic MLKL activation. Data are mean ± s.e.m.; statistical significance was determined using two-tailed unpaired Student's t-test (**b,f**) and one-way ANOVA with Šídák correction for multiple comparisons (**d**), with exact *P* values shown.

Supplementary Table 1 | Reagents and materials used in this study.

REAGENT OR RESOURCE	SOURCE	IDENTIFIER
**Antibodies**		
Armenian hamster anti-mouse CD3ε-PECy5 (145-2C11)	Thermo Fisher Scientific	15-0031-83; AB_468691
Armenian hamster anti-mouse CD3ε-PECy7 (145-2C11)	Thermo Fisher Scientific	25-0031-82; AB_469572
Armenian hamster anti-mouse CD48-AF700 (HM48-1)	BioLegend	103425; AB_10612754
Armenian hamster anti-mouse CD48-APCeF780 (HM48-1)	Thermo Fisher Scientific	47-0481-82; AB_2573962
Donkey anti-goat IgG (H+L)-AF488	Abcam	ab150129; AB_2687506
Goat anti-mouse IgG (H+L)-AF647	Thermo Fisher Scientific	A-21235; AB_2535804
Goat anti-mouse IgG-AF488	Thermo Fisher Scientific	A-11001; AB_2534069
Goat anti-mouse neogenin-1 (NEO-1)	Bio-Techne	AF1079; AB_2151002
Goat anti-rabbit IgG (H+L)-AF594	Thermo Fisher Scientific	A-11012; AB_2534079
Goat anti-rabbit IgG-5nm gold	BBI Solutions	EM GAR5/1; AB_1769142
Mouse anti-mouse CD45.1-APCeF780 (A20)	Thermo Fisher Scientific	47-0453-82; AB_1582228
Mouse anti-mouse CD45.2-BV786 (104)	Thermo Fisher Scientific	417-0454-82; AB_2929112
Mouse anti-mouse CD45.2-FITC (104)	Thermo Fisher Scientific	11-0454-85; AB_465062
Mouse anti-mouse COX-IV (3C7D2)	Proteintech	60251-1-Ig; AB_2881372
Mouse anti-mouse MLKL (E7V4W)	Cell Signaling Technology	26539; AB_3608292
Mouse anti-mouse γH2AX (JBW301)	Millipore	05-636
Rabbit anti-FLAG (DYKDDDDK)	Thermo Fisher Scientific	740001; AB_2610628
Rabbit anti-mouse GPR183-FITC	Thermo Fisher Scientific	AGR-063; AB_2925069
Rabbit anti-mouse p-MLKL (S345) (D6E3G)	Cell Signaling Technology	37333; AB_2799112
Rat anti-mouse B220-AF700 (RA3-6B2)	Thermo Fisher Scientific	56-0452-82; AB_891458
Rat anti-mouse B220-BV605 (RA3-6B2)	Thermo Fisher Scientific	406-0452-82; AB_2937168
Rat anti-mouse B220-PECy5 (RA3-6B2)	Thermo Fisher Scientific	15-0452-83; AB_468756

Rat anti-mouse c-Kit-APC (2B8)	Thermo Fisher Scientific	17-1171-83; AB_469431
Rat anti-mouse CD150-BV650 (TC15-12F12.2)	BioLegend	115932; AB_2715765
Rat anti-mouse CD150-PE (TC15-12F12.2)	BioLegend	115904; AB_313683
Rat anti-mouse CD34-FITC (RAM34)	Thermo Fisher Scientific	11-0341-85; AB_465022
Rat anti-mouse CD4-PECy5 (GK1.5)	Thermo Fisher Scientific	15-0041-83; AB_468696
Rat anti-mouse CD41-BV421 (MWReg30)	BioLegend	133911; AB_10960744
Rat anti-mouse CD41-FITC (MWReg30)	BD Biosciences	553848; AB_395085
Rat anti-mouse CD5-PECy5 (53-7.3)	BioLegend	100610; AB_312739
Rat anti-mouse CD8α-PECy5 (53-6.7)	Thermo Fisher Scientific	15-0081-83; AB_468707
Rat anti-mouse EPCR-PE (eBio1560)	Thermo Fisher Scientific	12-2012-82; AB_914317
Rat anti-mouse FcγR (93)	BioLegend	101302; AB_312801
Rat anti-mouse FcγR-BV510 (93)	BioLegend	101333; AB_2563692
Rat anti-mouse Flk2-Bio (A2F10)	Thermo Fisher Scientific	13-1351-85; AB_466600
Rat anti-mouse Flk2-BV421 (A2F10)	BioLegend	135315; AB_2571919
Rat anti-mouse Gr-1-eF450 (RB6-8C5)	Thermo Fisher Scientific	48-5931-82; AB_1548788
Rat anti-mouse Gr-1-PECy5 (RB6-8C5)	Thermo Fisher Scientific	15-5931-83; AB_468814
Rat anti-mouse IL-7Rα-APCCy7 (A7R34)	BioLegend	135039; AB_2566160
Rat anti-mouse IL-7Rα-PE (A7R34)	Thermo Fisher Scientific	12-1271-83; AB_465845
Rat anti-mouse Ki-67-FITC (SolA15)	Thermo Fisher Scientific	11-5698-82; AB_11151330
Rat anti-mouse Mac-1-APC (M1/70)	Thermo Fisher Scientific	17-0112-83; AB_469344
Rat anti-mouse Mac-1-Bio (M1/70)	Tonbo Biosciences	30-0112; AB_2621639
Rat anti-mouse Mac-1-PECy5 (M1/70)	Thermo Fisher Scientific	15-0112-83; AB_468715
Rat anti-mouse P-selectin-PE (RMP-1)	BioLegend	161204; AB_2876576

Rat anti-mouse Sca-1-Bio (D7)	BioLegend	108104; AB_313340
Rat anti-mouse Sca-1-PECy7 (D7)	Thermo Fisher Scientific	25-5981-82; AB_469669
Rat anti-mouse Ter119-PECy5 (TER-119)	Thermo Fisher Scientific	15-5921-83; AB_468811
**Chemicals, peptides, and recombinant proteins**		
2-deoxy-D-glucose	Tokyo Chemical Industry Co., Ltd.	D0051
5-FU	Kyowa Kirin	N/A
Annexin V-FITC	BioLegend	640945; AB_2629519
Antimycin	Sigma-Aldrich	A8674
Bafilomycin A1	Cayman Chemical	11038
CCCP	Abcam	ab141229
DAPI	Thermo Fisher Scientific	D1306
FCCP	Sigma-Aldrich	C2920
Histopaque-1119	Sigma-Aldrich	11191-6X100ML
LPS	InvivoGen	tlrl-eblps
MitoTracker Green	Thermo Fisher Scientific	M7514
Oligomycin	Cell Signaling Technology	9996L
pIC	Cytiva	27473201
Polyvinyl alcohol	Japan Vam & Poval	PE-05JPS
Propidium iodide	BD Biosciences	556463; AB_2869075
Recombinant human TPO	BioLegend	763706
Recombinant mouse MLKL C-terminal domain (179-462)	This study	N/A
Recombinant mouse MLKL N-terminal domain (1-169)	This study	N/A
Recombinant mouse SCF	BioLegend	579706
Recombinant mouse TNF-α	Genentech	N/A
Recombinant mouse TPO	PeproTech	315-14
Recombinant N-terminal 3×FLAG-tagged mouse MLKL N-terminal domain (1-169)	This study	N/A
Rotenone	Sigma-Aldrich	R8875
Streptavidin-BV605	BioLegend	405229
SYTOX Blue Dead Cell Stain	Thermo Fisher Scientific	S34857
TMRM	Thermo Fisher Scientific	I34361
UH15-38	Bio-Techne	8104/50
**Critical commercial assays**		
Anti-Biotin MicroBeads	Miltenyi Biotec	130-090-485; AB_244365
Bio-Plex Pro Mouse Cytokine 23-plex Assay	Bio-Rad	M60009RDPD

CellROX Green	Thermo Fisher Scientific	C10492
Cytofix/Cytoperm Fixation/Permeablization Kit	BD Biosciences	554714; AB_2869008
DALGreen	Dojindo	D675-10
Duolink In Situ Detection Reagents GREEN	Sigma-Aldrich	DUO92014
Duolink In Situ Orange Starter Kit mouse/Rabbit	Sigma-Aldrich	DUO92102
FuGENE HD	Promega	E2312
JC-1 MitoMP Detection Kit	Dojindo	349-09401
Mitochondria Isolation Kit for Cultured Cells (with Dounce Homogenizer)	Abcam	ab110171
Mouse c-Kit microbeads	Miltenyi Biotec	130-091-224; AB_2753213
NEBNext Ultra DNA Library Prep Kit	New England BioLabs	E7370L
ProLong Glass Antifade Mountant	Thermo Fisher Scientific	P36980
ProLong Gold Antifade Mountant with DAPI	Thermo Fisher Scientific	P36935
RNeasy Plus Micro Kit	QIAGEN	74034
Seahorse XF DMEM medium, pH 7.4	Agilent Technologies	103575-100
SMART-Seq HT Kit	Takara Bio	634437
ViroMag R/L	OZ biosciences	RL-40200
**Deposited data**		
ATAC-seq of WT and *Mlkl*$^{-/-}$ HSCs ± ageing	This study	GSE285111
RNA-seq of WT and *Mlkl*$^{-/-}$ HSCs ± ageing	This study	GSE285111
**Experimental models: Cell lines**		
293GPG: pMY-RUNX1S291fs-IRES-GFP	This study	N/A
Plat-E	Morita et al. Gene Ther 2000	CVCL_B488
**Experimental models: Organisms/strains**		
Mouse: C57BL/6-CD45.1	Sankyo-Labo Service	N/A
Mouse: C57BL/6-CD45.2	Japan SLC	C57BL/6JmsSlc
Mouse: C57BL/6J	Jackson Laboratories	IMSR_JAX:000664
Mouse: *Mlkl*$^{-/-}$	Lin et al. Nature 2016	N/A
Mouse: *Mlkl*^{SA2}	Jackson Laboratories	IMSR_JAX:039024
Mouse: *Ripk3*$^{-/-}$ SMART-Tg	Murai et al. Commun Biol 2022	N/A
Mouse: SMART-Tg	Murai et al. Commun Biol 2022	N/A
**Recombinant DNA**		
pMY-3×FLAG-MLKL-IRES-GFP	This study	N/A
pMY-IRES-GFP	Kitamura et al. Exp Hematol 2003	Addgene_163361
**Software and algorithms**		
Bcl2fastq v2.20	Illumina	SCR_015058
Bedtools v2.31.0	Quinlan and Hall. Bioinformatics 2010	SCR_006646

Bowtie2 v2.5.3	Langmead and Salzberg. Nat Methods 2012	SCR_016368
edgeR v3.30.3	Robinson et al. Bioinformatics 2010	SCR_012802
FastQC v0.12.0	Babraham Bioinformatics	SCR_014583
FlowJo v10.10.0	BD Biosciences	SCR_008520
Gene set enrichment analysis v4.3.0	Subramanian et al. Proc Natl Acad Sci U S A 2005	SCR_003199
HISAT2 v2.2.1	Kim et al. Nat Methods 2015	SCR_015530
Image Lab software v6.1.0	Bio-Rad	SCR_014210
ImageJ v1.53c	Schneider et al. Nat Methods 2012	SCR_003070
MACS2 v2.2.7.1	Zhang et al. Genome Biol 2008	SCR_013291
Morpheus	Broad Institute	SCR_017386
NIS-Elements Viewer v4.11.0	Nikon	SCR_014329
Prism v10.4.0	GraphPad	SCR_002798
R v4.0.2	Dessau and Pippere. Ugeskr Laeger 2008	SCR_001905
Seahorse Analytics v1.0.0-520	Agilent	SCR_019545
StringTie v2.2.3	Pertea et al. Nat Biotechnol 2015	SCR_016323
