# Supplementary material for: Non-necroptotic MLKL function damages mitochondria and promotes hematopoietic stem cell aging
Source: Nat Commun. 2026 Apr 6;17:2798. doi: 10.1038/s41467-026-71060-4 (PMC13053712; doi:10.1038/s41467-026-71060-4)
Supplement: Supplementary file 2 — Description of Additional Supplementary Files [file 41467_2026_71060_MOESM2_ESM.pdf]

Description of Additional Supplementary Files

File name: Supplementary Data 1
Description: DEGs between young WT and young *Mlkl*$^{-/-}$ BM HSCs.

File name: Supplementary Data 2
Description: DEGs between young WT and aged WT BM HSCs.

File name: Supplementary Data 3
Description: DEGs betwen aged WT and aged *Mlkl*$^{-/-}$ BM HSCs.

File name: Supplementary Data 4
Description: DEGs between young *Mlkl*$^{-/-}$ and aged *Mlkl*$^{-/-}$ BM HSCs.

File name: Supplementary Data 5
Description: DARs between young WT and young *Mlkl*$^{-/-}$ BM HSCs.

File name: Supplementary Data 6
Description: DARs between young WT and aged WT BM HSCs.

File name: Supplementary Data 7
Description: DARs between aged WT and aged *Mlkl*$^{-/-}$ BM HSCs.

File name: Supplementary Data 8
Description: DARs between young *Mlkl*$^{-/-}$ and aged *Mlkl*$^{-/-}$ BM HSCs.
