# Supplementary material for: Non-necroptotic MLKL function damages mitochondria and promotes hematopoietic stem cell aging
Source: Nat Commun. 2026 Apr 6;17:2798. doi: 10.1038/s41467-026-71060-4 (PMC13053712; doi:10.1038/s41467-026-71060-4)
Supplement: Supplementary file 4 — Reporting Summary [file 41467_2026_71060_MOESM4_ESM.pdf]

nature portfolio

Corresponding author(s): Masayuki Yamashita

Last updated by author(s): Feb 10, 2026

Reporting Summary

Nature Portfolio wishes to improve the reproducibility of the work that we publish. This form provides structure for consistency and transparency in reporting. For further information on Nature Portfolio policies, see our Editorial Policies and the Editorial Policy Checklist.

Statistics

For all statistical analyses, confirm that the following items are present in the figure legend, table legend, main text, or Methods section.

n/a	Confirmed	
☐	☒	The exact sample size (*n*) for each experimental group/condition, given as a discrete number and unit of measurement
☐	☒	A statement on whether measurements were taken from distinct samples or whether the same sample was measured repeatedly
☐	☒	The statistical test(s) used AND whether they are one- or two-sided<br>*Only common tests should be described solely by name; describe more complex techniques in the Methods section.*
☒	☐	A description of all covariates tested
☐	☒	A description of any assumptions or corrections, such as tests of normality and adjustment for multiple comparisons
☐	☒	A full description of the statistical parameters including central tendency (e.g. means) or other basic estimates (e.g. regression coefficient) AND variation (e.g. standard deviation) or associated estimates of uncertainty (e.g. confidence intervals)
☐	☒	For null hypothesis testing, the test statistic (e.g. *F*, *t*, *r*) with confidence intervals, effect sizes, degrees of freedom and *P* value noted<br>*Give P values as exact values whenever suitable.*
☒	☐	For Bayesian analysis, information on the choice of priors and Markov chain Monte Carlo settings
☒	☐	For hierarchical and complex designs, identification of the appropriate level for tests and full reporting of outcomes
☒	☐	Estimates of effect sizes (e.g. Cohen's *d*, Pearson's *r*), indicating how they were calculated

Our web collection on statistics for biologists contains articles on many of the points above.

Software and code

Policy information about availability of computer code

Data collection	Flow cytometry data were collected on FACS Aria IIIu, Celesta, or LSR Fortessa (Becton Dickinson). Confocal and super-resolution images were acquired on N-SIM super-resolution confocal microscope with a 100× objective (Nikon). TEM and immuno-EM images were collected on JEM-1400Flash electron microscope (JEOL). Cytokine data were collected on Bio-Plex 200 analyzer (Bio-Rad). Seahorse assay data were collected using Seahorse XFe96 Extracellular Flux Analyzer (Agilent).
Data analysis	Statistical analyses were performed in GraphPad Prism (v10.4.0) and R (v4.0.2). RNA-seq: FastQC (v0.12.0), bcl2fastq (v2.20), HISAT2 (v2.2.1), StringTie (v2.2.3), edgeR (v3.30.3); GSEA (v4.3.0). ATAC-seq: Bowtie2 (v2.5.3), MACS2 (v2.2.7.1), bedtools (v2.31.0), edgeR. Flow cytometry: FlowJo (v10.10.0). Confocal images were visualized and analyzed with NIS-Elements Viewer (v4.11.0) and ImageJ (v1.53c). Morpheus was used for heatmaps. Seahorse data were processed with Seahorse Analytics (v1.0.0-520).

For manuscripts utilizing custom algorithms or software that are central to the research but not yet described in published literature, software must be made available to editors and reviewers. We strongly encourage code deposition in a community repository (e.g. GitHub). See the Nature Portfolio guidelines for submitting code & software for further information.

Data

Policy information about availability of data

All manuscripts must include a data availability statement. This statement should provide the following information, where applicable:

- Accession codes, unique identifiers, or web links for publicly available datasets
- A description of any restrictions on data availability
- For clinical datasets or third party data, please ensure that the statement adheres to our policy

All source data supporting the findings are provided with this paper. Bulk RNA-seq data and ATAC-seq data from have been deposited at Gene Expression Omnibus and are publicly available under the accession number GSE285111.

Research involving human participants, their data, or biological material

Policy information about studies with human participants or human data. See also policy information about sex, gender (identity/presentation), and sexual orientation and race, ethnicity and racism.

Reporting on sex and gender	n/a
Reporting on race, ethnicity, or other socially relevant groupings	n/a
Population characteristics	n/a
Recruitment	n/a
Ethics oversight	n/a

Note that full information on the approval of the study protocol must also be provided in the manuscript.

Field-specific reporting

Please select the one below that is the best fit for your research. If you are not sure, read the appropriate sections before making your selection.

☒ Life sciences ☐ Behavioural & social sciences ☐ Ecological, evolutionary & environmental sciences

For a reference copy of the document with all sections, see nature.com/documents/nr-reporting-summary-flat.pdf

Life sciences study design

All studies must disclose on these points even when the disclosure is negative.

Sample size	No statistical methods were used to predetermine sample size. Sample sizes were chosen based on prior experience with similar hematopoietic stem cell experiments and are sufficient to detect biologically meaningful differences. Exact n values for each experiment are indicated in figure legends.
Data exclusions	A few mice that developed idiopathic tumors were excluded from analyses to ensure evaluation of normal hematopoiesis. For transplantation experiments, outliers and non-engrafted animals were excluded based on the exclusion criteria described in the Methods section.
Replication	All experiments were performed with multiple replicates. The number of replicates (n) and independent experiments performed were detailed in the figure legends.
Randomization	Samples were randomly allocated to experimental groups.
Blinding	Blinding was not feasible because animals were grouped by genotype. However, objective outcome measures and standardized protocols were used to minimize potential bias.

Reporting for specific materials, systems and methods

We require information from authors about some types of materials, experimental systems and methods used in many studies. Here, indicate whether each material, system or method listed is relevant to your study. If you are not sure if a list item applies to your research, read the appropriate section before selecting a response.

Materials & experimental systems

n/a	Involved in the study
☐	☒ Antibodies
☐	☒ Eukaryotic cell lines
☒	☐ Palaeontology and archaeology
☐	☒ Animals and other organisms
☒	☐ Clinical data
☒	☐ Dual use research of concern
☒	☐ Plants

Methods

n/a	Involved in the study
☒	☐ ChIP-seq
☐	☒ Flow cytometry
☒	☐ MRI-based neuroimaging

Antibodies

Antibodies used

Armenian hamster anti-mouse CD3ε-PECy5 (145-2C11), Thermo Fisher Scientific, 15-0031-83; AB_468691
Armenian hamster anti-mouse CD3ε-PECy7 (145-2C11), Thermo Fisher Scientific, 25-0031-82; AB_469572
Armenian hamster anti-mouse CD48-AF700 (HM48-1), BioLegend, 103425; AB_10612754
Armenian hamster anti-mouse CD48-APCeF780 (HM48-1), Thermo Fisher Scientific, 47-0481-82; AB_2573962
Donkey anti-goat IgG (H+L)-AF488, Abcam, ab150129; AB_2687506
Goat anti-mouse IgG (H+L)-AF647, Thermo Fisher Scientific, A-21235; AB_2535804
Goat anti-mouse IgG-AF488, Thermo Fisher Scientific, A-11001; AB_2534069
Goat anti-mouse neogenin-1 (NEO-1), Bio-Techne, AF1079; AB_2151002
Goat anti-rabbit IgG (H+L)-AF594, Thermo Fisher Scientific, A-11012; AB_2534079
Goat anti-rabbit IgG-5nm gold, BBI Solutions, EM GAR5/1; AB_1769142
Mouse anti-mouse CD45.1-APCeF780 (A20), Thermo Fisher Scientific, 47-0453-82; AB_1582228
Mouse anti-mouse CD45.2-BV786 (104), Thermo Fisher Scientific, 417-0454-82; AB_2929112
Mouse anti-mouse CD45.2-FITC (104), Thermo Fisher Scientific, 11-0454-85; AB_465062
Mouse anti-mouse COX-IV (3C7D2), Proteintech, 60251-1-Ig; AB_2881372
Mouse anti-mouse MLKL (E7V4W), Cell Signaling Technology, 26539; AB_3608292
Mouse anti-mouse γH2AX (JBW301), Millipore, 05-636
Rabbit anti-FLAG (DYKDDDDK), Thermo Fisher Scientific, 740001; AB_2610628
Rabbit anti-mouse GPR183-FITC, Thermo Fisher Scientific, AGR-063; AB_2925069
Rabbit anti-mouse p-MLKL (S345) (D6E3G), Cell Signaling Technology, 37333; AB_2799112
Rat anti-mouse B220-AF700 (RA3-6B2), Thermo Fisher Scientific, 56-0452-82; AB_891458
Rat anti-mouse B220-BV605 (RA3-6B2), Thermo Fisher Scientific, 406-0452-82; AB_2937168
Rat anti-mouse B220-PECy5 (RA3-6B2), Thermo Fisher Scientific, 15-0452-83; AB_468756
Rat anti-mouse c-Kit-APC (2B8), Thermo Fisher Scientific, 17-1171-83; AB_469431
Rat anti-mouse CD150-BV650 (TC15-12F12.2), BioLegend, 115932; AB_2715765
Rat anti-mouse CD150-PE (TC15-12F12.2), BioLegend, 115904; AB_313683
Rat anti-mouse CD34-FITC (RAM34), Thermo Fisher Scientific, 11-0341-85; AB_465022
Rat anti-mouse CD4-PECy5 (GK1.5), Thermo Fisher Scientific, 15-0041-83; AB_468696
Rat anti-mouse CD41-BV421 (MWReg30), BioLegend, 133911; AB_10960744
Rat anti-mouse CD41-FITC (MWReg30), BD Biosciences, 553848; AB_395085
Rat anti-mouse CD5-PECy5 (53-7.3), BioLegend, 100610; AB_312739
Rat anti-mouse CD8α-PECy5 (53-6.7), Thermo Fisher Scientific, 15-0081-83; AB_468707
Rat anti-mouse EPCR-PE (eBio1560), Thermo Fisher Scientific, 12-2012-82; AB_914317
Rat anti-mouse FcγR (93), BioLegend, 101302; AB_312801
Rat anti-mouse FcγR-BV510 (93), BioLegend, 101333; AB_2563692
Rat anti-mouse Flk2-Bio (A2F10), Thermo Fisher Scientific, 13-1351-85; AB_466600
Rat anti-mouse Flk2-BV421 (A2F10), BioLegend, 135315; AB_2571919
Rat anti-mouse Gr-1-eF450 (RB6-8C5), Thermo Fisher Scientific, 48-5931-82; AB_1548788
Rat anti-mouse Gr-1-PECy5 (RB6-8C5), Thermo Fisher Scientific, 15-5931-83; AB_468814
Rat anti-mouse IL-7Rα-APCCy7 (A7R34), BioLegend, 135039; AB_2566160
Rat anti-mouse IL-7Rα-PE (A7R34), Thermo Fisher Scientific, 12-1271-83; AB_465845
Rat anti-mouse Ki-67-FITC (SolA15), Thermo Fisher Scientific, 11-5698-82; AB_11151330
Rat anti-mouse Mac-1-APC (M1/70), Thermo Fisher Scientific, 17-0112-83; AB_469344
Rat anti-mouse Mac-1-Bio (M1/70), Tonbo Biosciences, 30-0112; AB_2621639
Rat anti-mouse Mac-1-PECy5 (M1/70), Thermo Fisher Scientific, 15-0112-83; AB_468715
Rat anti-mouse P-selectin-PE (RMP-1), BioLegend, 161204; AB_2876576
Rat anti-mouse Sca-1-Bio (D7), BioLegend, 108104; AB_313340
Rat anti-mouse Sca-1-PECy7 (D7), Thermo Fisher Scientific, 25-5981-82; AB_469669
Rat anti-mouse Ter119-PECy5 (TER-119), Thermo Fisher Scientific, 15-5921-83; AB_468811
See Method section for antibody amount/dilution information.

Validation

All primary antibodies were commercially available and validated by the manufacturers for the indicated applications. Specificity of immunofluorescent signals was confirmed using knockout cells as shown in Fig. 6c, 6d, Supplementary Fig. 3m, and 8b.

Eukaryotic cell lines

Policy information about cell lines and Sex and Gender in Research

Cell line source(s)	Plat-E packaging cells were kindly provided by Yosuke Tanaka (the Institute of Medical Science, University of Tokyo) and 293GPG packaging cells were kindly provided by Goro Sashida (Kumamoto University).
Authentication	Cell lines were not independently authenticated, as they were obtained from reliable sources and used solely for standard retroviral packaging.
Mycoplasma contamination	All cell lines tested were negative for mycoplasma contamination
Commonly misidentified lines (See ICLAC register)	n/a

Animals and other research organisms

Policy information about studies involving animals; ARRIVE guidelines recommended for reporting animal research, and Sex and Gender in Research

Laboratory animals	All animals used in this study are described in the Method section and listed in Supplementary Table 1.
Wild animals	No wild animals were used in this study.
Reporting on sex	Both male and female animals were used indiscriminately in this study
Field-collected samples	No field-collected samples were used in this study.
Ethics oversight	All animal experiments comply with all relevant ethical regulations and were performed in accordance with protocols approved by the Animal Care and Use Committee at the Institute of Medical Science, University of Tokyo (protocol number: PA19-07) and the St. Jude Children's Research Hospital Institutional Animal Care and Use Committee (protocol number: 3252).

Note that full information on the approval of the study protocol must also be provided in the manuscript.

Plants

Seed stocks	n/a
Novel plant genotypes	n/a
Authentication	n/a

Flow Cytometry

Plots

Confirm that:

☒ The axis labels state the marker and fluorochrome used (e.g. CD4-FITC).

☒ The axis scales are clearly visible. Include numbers along axes only for bottom left plot of group (a 'group' is an analysis of identical markers).

☒ All plots are contour plots with outliers or pseudocolor plots.

☒ A numerical value for number of cells or percentage (with statistics) is provided.

Methodology

Sample preparation	Single-cell suspensions were prepared from mouse bone marrow, spleen, and peripheral blood in PBS containing 2% heat-inactivated FBS. Red blood cells were lysed with ACK buffer, and cells were stained with fluorochrome-conjugated antibodies before flow cytometric analysis. Detailed staining protocols are described in the Methods section.
Instrument	Flow cytometric data were acquired using BD FACSCelesta and BD FACSAria III (BD Biosciences) at the Institute of Medical Science, the University of Tokyo, and BD LSRFortessa (BD Biosciences) at St. Jude Children's Research Hospital.

Software	Flow cytometry data were analyzed using FlowJo software (v10.10.0; BD Biosciences).
Cell population abundance	Cell population frequencies were determined by flow cytometry, and absolute cell numbers were calculated based on viable cell counts obtained with a Vi-CELL analyzer (Beckman Coulter).
Gating strategy	Debris were excluded based on FSC-A, and dead cells (PI+) were excluded except for Annexin V dying/dead cell analysis. Lin– cells were defined based on the backgating of Sca-1+/c-Kit+ cells. Gating strategies to enrich individual immature hematopoietic populations are provided in Supplementary Fig. 1a. Boundaries between positive and negative were defined based on appropriate negative control staining. FRET/CFP-high cells were identified based on increase of the FRET/CFP ratio and decrease of CFP signal, usually corresponding to the top 10% of the FRET/CFP ratio in control samples.

☒ Tick this box to confirm that a figure exemplifying the gating strategy is provided in the Supplementary Information.
